# Si-Wu-Tang ameliorates fibrotic liver injury via modulating intestinal microbiota and bile acid homeostasis

**DOI:** 10.1186/s13020-021-00524-0

**Published:** 2021-11-04

**Authors:** Xiaoyong Xue, Jianzhi Wu, Mingning Ding, Feng Gao, Fei Zhou, Bing Xu, Mingjun Lu, Jun Li, Xiaojiaoyang Li

**Affiliations:** 1 School of Life Sciences, Beijing University of Chinese Medicine, 11 Bei San Huan Dong Lu, Beijing, 100029 China; 2 School of Chinese Materia Medica, Beijing University of Chinese Medicine, 11 Bei San Huan Dong Lu, Beijing, 100029 China; 3 Gynecology Department, Dongzhimen Hospital, Beijing University of Chinese Medicine, Beijing, 100700 China

**Keywords:** Si-Wu-Tang, Fibrotic liver injury, Bile acid, Farnesoid X receptor, Intestinal microbiota

## Abstract

**Background:**

Fibrotic liver injury is a progressive scarring event, which may permanently affect liver function and progress into devastating end-stage liver diseases due to the absence of effective therapies. Si-Wu-Tang (SWT), a traditional Chinese medicine formula used in clinic to treat gynecological disorders for centuries, has been investigated in recent preliminary findings for its role in alleviating chronic liver diseases. Here we aim to elucidate the therapeutic effects and possible mechanisms of SWT against fibrotic liver injury.

**Methods:**

UHPLC-MS/MS was performed to investigate the chemical characterization of SWT. After intragastrically administered with carbon tetrachloride (CCl_4_) every 3 days for 1-week, C57BL/6 mice were orally administered with SWT (5.2, 10.4 and 20.8 g/kg) once daily for 3 weeks along with CCl_4_ challenge. Liver function was determined by the measurement of serum biomarkers, hematoxylin and eosin (H&E) and Masson’s trichrome staining. Intestinal inflammatory infiltration and the disruption of intestinal barrier were examined by H&E and E-cadherin immunohistochemical staining. The microbial composition of intestinal content was determined by 16S rRNA sequencing. Serum bile acids (BAs) profiling was analyzed by LC–MS/MS. Simultaneously, the expression of genes of interest was determined by qPCR and western blot.

**Results:**

SWT exhibited remarkable therapeutic effects on CCl_4_-induced liver fibrosis, as indicated by improved collagen accumulation in livers, intestinal barrier injury and hepatic and intestinal inflammatory response. Results of 16S rRNA sequencing revealed that SWT treatment strikingly restructured intestinal microbiota in fibrotic mice by increasing the relative abundances of *Bacteroides* and *Lachnoclostridium* and decreasing the relative abundances of *Alistipes* and *Rikenellaceae*. UHPLC-MS/MS data suggested that SWT altered the composition of BAs in circulation as evidenced by increased unconjugated BAs like cholic acid and chenodeoxycholic acid but decreased conjugated BAs including taurocholic acid and taurodeoxycholic acid, compared to that in CCl_4_ mice. Notably, SWT efficiently improved the imbalance of BA homeostasis in livers caused by CCl_4_ via activating farnesoid X receptor (FXR)-fibroblast growth factor 15 enterohepatic and FXR-small heterodimer partner hepatic pathways.

**Conclusion:**

SWT decreased inflammatory response, reconstructed gut microbiota-mediated BA homeostasis as well as activated FXR pathways, which eventually protected against CCl_4_-induced fibrotic liver injury.

**Supplementary Information:**

The online version contains supplementary material available at 10.1186/s13020-021-00524-0.

## Introduction

Chronic inflammation or abnormal bile acid (BA) accumulation in response to persistent exogenous stimuli or damage progressively result in fibrotic liver injury, characterized by the excessive extracellular matrix (ECM) deposition and scarring formation. If left untreated, liver fibrosis may progress to the point of cirrhosis and even hepatocellular carcinoma, which permanently affect liver function and cause a serious threat to human health [1]. Substantial evidence supported a role of persistent cholestasis, viral infection, alcohol abuse, as well as inflammatory cytokines in the initiation and progression of fibrotic liver injury. BAs irreplaceably modulate lipid, glucose and energy homeostasis and represent as one of the important circuits in the bidirectional interplay between liver and intestinal microbiome [2]. Notably, high prevalence of increased serum BAs, bloody diarrhea, shortening and widening of the microvilli and the decreased villous/crypt ratio were noted in patients with hepatic fibrotic injury [3] and mouse studies have recently indicated a physiological correlation between the disorder of BA-related liver fibrosis and the destruction of intestinal microbiota [4].

Once hepatocyte injury, the size and composition of BA pool circulating in blood and gastrointestinal (GI) tract are changed. After being released into the intestine, the altered BAs regulate the growth of BA-sensitive bacteria and thus reshape the gut microbiome community [5]. On the one hand, intestinal dysbiosis and subsequent disruption of intestinal barrier allow the translocation of inflammatory factors, pathogens and pathogen-associated molecular patterns (PAMPs) into the portal vein and systemic circulations, which further interact with hepatic stellate cells (HSCs) and immune cells in livers and dysregulate hepatic functions. On the other hand, gut microbiota contributes to the deconjugation, oxidation, epimerization, 7-dehydroxylation, esterification and desulfation of BAs, which serve as imperative gateway reactions in BA metabolism and cause a vicious cycle of hepatoenteral injury [5, 6]. The increased BAs are sensed by intestinal farnesoid X receptor (FXR) to induce mouse fibroblast growth factor 15 (FGF15, FGF19 in humans), which binds to FGF receptor 4 (FGFR4) on hepatocytes and feedback inhibits BA biogenesis [7]. Meanwhile, BAs in GI tract, mainly conjugated bile acids (CBAs) are re-absorbed at the distal ileum into enterocytes and returned to the liver, which may inhibit several nuclear receptors including FXR and cause excessive retention of BAs in the liver [8]. Subsequently, accumulated CBAs, such as taurocholic acid (TCA), were reported to upregulate the hepatic expressions of fibrotic markers and inflammatory factors, leading to HSC activation and liver fibrosis [9, 10].

Currently, colchicine, methotrexate and ursodeoxycholic acid (UDCA) are recommended as first-line therapeutics for liver fibrosis and complications [1]. Unfortunately, the clinical response rate to these therapies is low and is inconsistent among different clinal trials. Also, a considerable number of patients taking these medicines continuously suffer adverse effects. Considering the crucial roles of BA signaling and microbial interactions, medicines targeting the gut-liver axis may become promising options for the treatment of fibrotic liver injury. Nowadays, a modified FXR agonist obeticholic acid (OCA) and another engineered FGF19 analogue NGM282 have been reported to regulate BA metabolism, alleviate gut dysbiosis and improve fibrotic liver injury in clinical trials [11, 12]. Further investigation of therapeutic targets and the exploration of novel medications aiming for liver-gut axis are of both basic science and clinical relevance.

Si-Wu-Tang (SWT), a traditional Chinese medicine (TCM) formula, was first documented in *Taiping Huimin Heji Jufang*, recorded in the Song Dynasty. It consists of *Radix Rehmanniae Praeparata* (RRP, shu di huang), *Radix Paeoniae Alba* (RPA, bai shao), *Radix Angelica Sinensis* (RAS, dang gui), *Rhizoma Ligusticum Chuanxiong* (RLC, chuan xiong). SWT has been traditionally used for relieving gynecological disorders such as menstrual discomfort and climacteric syndrome [13]. Several clinical and preclinical studies reported that SWT effectively decreased gallstones, improved lipid metabolism and relived liver injury [14, 15]. We recently documented that the combination of RAS and RLC effectively ameliorated fibrotic liver injury by decreasing BA accumulation and repressing the production of inflammatory cytokines [16]. Bioactive ingredients of RPA and RRP exhibited hepatoprotective effects as well [17]. However, the underlying mechanism of SWT in treating fibrotic liver injury remains elusive yet and demands urgent clarification.

In this study, we aim to explore the protective effects of SWT in a carbon tetrachloride (CCl_4_)-induced fibrotic mouse model and further elucidate possible mechanisms. Our results demonstrated that SWT significantly alleviated fibrotic liver injury via restructuring intestinal microbiota, regulating BA homeostasis and activating FXR-FGF15 enterohepatic and FXR-small heterodimer partner (SHP) hepatic pathways. Collectively, our findings strongly suggest that SWT may serve as a promising therapeutic option for the treatment of fibrotic liver injury and its complications by regulating the liver-gut axis.

## Materials and methods

### Materials

CCl_4_ and other chemicals were purchased from Sigma-Aldrich (St. Louis, MO). Formaldehyde (PFA) and bovine serum albumin (BSA) were obtained from Solarbio Technology Co., Ltd. (Beijing, China). iQ™ SYBR Green Supermix was purchased from Bio-Rad (Hercules, CA). Antibody against COLLAGEN (COL1) (ab34710) was purchased from Abcam (MA, USA). Antibodies against FIBRONECTIN (FN) (15613-1-AP) and E-Cadherin (ECAD) (20874-1-AP) were purchased from Proteintech Group, Inc. (Rosemont, USA). Antibody against FXR (SC-25309) was purchased from Santa Cruz Biotechnology (Santa Cruz, USA). Antibodies against alpha-smooth muscle actin (α-SMA) (19245S) and β-ACTIN (4970S) were obtained from Cell Signaling Technology (Danvers, USA).

### Preparation of SWT

SWT was composed of RRP, RPA, RAS and RLC with a ratio of 1:1:1:1. Commercial herbal products of RRP, RPA, RAS and RLC were all purchased from Beijing Tongrentang (Group) Co. LTD (China) and had passed quality verification by the National Medical Products Administration (China). All purchased herbal products were then authenticated by Dr. Bing Xu from the School of Materia Medica, Beijing University of Chinese Medicine. All herbal products were sliced and then extracted with eightfold volumes of distilled water by the condensation reflux method for two times. The two fractions of water extract were combined and concentrated with a rotary evaporator at 45 °C to a final concentration of about 1 g/ml and filtered twice with 100 μm mesh and 0.45 μm filter, respectively. Finally, prepared SWT was aliquoted into sterile tubes and stored at − 80 °C to avoid freeze/thaw cycles.

### UHPLC-MS/MS for the identification of ingredients in SWT

Ultra-high-performance liquid chromatography (UHPLC)-UV analysis was carried on an UltiMate 3000 liquid chromatograph (Thermo Scientific, Sunnyvale, CA, USA) equipped with auto sampler, degasser, diode-array detector and column compartment. The separation of SWT decoction was performed using an analytical column Waters ACQUITYTM UPLC BEH C18 column with column sizes of 2.1 mm × 100 mm and particle size of 1.7 μm (Waters Corporation; Milford, MA, USA). The mobile phase consisted of 0.1% formic acid (A) and acetonitrile (B). The separation was performed on the following gradient program: 2–4% B at 0–1 min, 4–10% B at 1–4 min, 10–12% B at 4–5 min, 12% B at 5–5.8 min, 12–20% B at 5.8–11 min, 20% B at 11–13 min, 20–40% B at 13–15 min, 40% B at 15–17 min, 40–60% B at 17–19 min, 60% B at 19–22 min, 60–90% B at 22–24 min, 90% B at 24–30 min. The column temperature was maintained at 25 °C and the injection volume was 3 μl with a flow rate of 0.3 ml/min during separation. The MS experiments were performed on a benchtop Q Exactive hybrid quadrupole-Orbitrap mass spectrometer (Thermo Scientific, Bremen, Germany) equipped with an ESI interface. The ionization parameters were set as follows: spray voltage 3.5 kV, heated capillary temperature of 320 °C and auxiliary gas heater temperature of 400 °C; sheath gas and auxiliary gas flow rates of 35 and 10 (arbitrary units). The mass spectrometry was programmed to perform in both positive and negative ionization mode and under the full scan analysis with mass range of m/z 100–1500.

### Animal studies

C57BL/6J mice (8 weeks old, male and female, 22–24 g) were purchased from SIBEIFU Biotechnology Co, Ltd. (Beijing, China). Mice were housed in a temperature-controlled room (22 ± 2 °C) with a humidity of 40 ± 10% and 12:12-h light/dark cycle and had free access to normal chow diet and sterile water. CCl_4_ was dissolved in olive oil (15% v/v, 1 ml/kg). After acclimatization for 7 days, mice were randomly divided into five groups (n = 8): (1) control group; (2) CCl_4_ group; (3) CCl_4_ + SWT low dose (L) group; (4) CCl_4_ + SWT medium dose (M) group; (5) CCl_4_ + SWT high dose (H) group. Groups (2)–(5) were intragastrically administered with CCl_4_ every 3 days for 4 weeks as previously described [16]. Groups (3)–(5) were intragastrically administered with SWT (L) (5.2 g/kg), SWT (M) (10.4 g/kg) and SWT (H) (20.8 g/kg) daily for 3 weeks after 1-week CCl_4_ administration. At the end of the experiment, mice were anesthetized with isoflurane and sacrificed to collect blood and livers. All animal studies and procedures were approved by the Institutional Animal Care and Use Committee of Beijing University of Chinese Medicine and were carried out in accordance with all guidelines and regulations.

### FGF15 ELISA assay

Serum FGF15 level was determined using FGF15 ELISA Kit (MEL154Mu, Wuhan USCN Business Co. Ltd) according to the manufacturer’s instructions. First, standards (0–1000 pg/ml) or testing samples were added to the microplate wells pre-coated with a biotin-conjugated antibody against FGF15. Next, HRP-avidin reagents and TMB substrates were added to these microplate wells, respectively. Then, the incubation was terminated with a stop solution and O.D. values were measured at a wavelength of 450 nm. Finally, the concentrations of serum FGF15 were calculated based on data from the standard curve.

### Measurement of serum BAs

BAs in mouse serum were analyzed quantitatively by LC–MS/MS analysis using AB Sciex Qtrap 5500 and Nexera UHPLC LC-30A (AB Sciex, USA). 42 of BA standards including cholic acid (CA), lithocholic acid (LCA), TCA, chenodeoxycholic acid (CDCA), deoxycholic acid (DCA), lithocholyltaurine (TLCA), hyocholic acid (HCA), hyodeoxycholic acid (HDCA) and other BAs used in this study were obtained from Sigma-Aldrich (St. Louis, MI). The chromatographic conditions were listed as follows: the column was a Phenomenex Kinetex C18 (2.1 mm × 100 mm, 2.6 µm; FLM Scientific Instrument, Guangzhou, China); mobile phase A was water containing 0.1% formic acid, and mobile phase B was methanol/acetonitrile/isopropanol (1/1/1) containing 0.1% formic acid. After setting to desired column temperature about 45 °C, the solvent gradient procedures changed according to the following conditions: from 0 to 5 min, 70% (A): 30% (B) to 60% (A): 40% (B); from 5 to 13 min, 60% (A): 40% (B) to 45% (A): 55% (B); from 13 to 17 min, 45% (A): 55% (B) to 20% (A): 80% (B); from 17 to 18 min, 20% (A): 80% (B) to 10% (A): 90% (B), from 18 to 20 min, 10% (A): 90% (B) to 65% (A): 35% (B) for equilibrating the systems. The serum sample injection volume was 5 μl and the flow rate was set to 0.3 ml/min. In addition, the sample mass spectrometer signals were collected using positive and negative ion scanning modes. Chromatographic separation was followed by negative ion electrospray ionization (ESI) tandem mass spectrometry in the multiple reaction monitoring (MRM) mode. The conditions of the mass spectrometer were as follows: ion-spray voltage, − 4.5 kV; curtain gas, 35 psi; collision gas, medium; source temperature, 450 °C; ion source gas 1, 55 psi; ion source gas 2, 55 psi.

### 16S ribosomal RNA gene sequencing and analysis

Bacterial DNA was extracted from intestinal contents using a MagPure Soil DNA LQ Kit (Magen, Guangdong, China) in accordance with the manufacturer’s protocols. Then, PCR amplification of the V3–V4 hypervariable regions of the bacterial 16S rRNA gene was performed using universal primers (343F—5′-TACGGRAGGCAGCAG-3′, 798R—5′-AGGGTATCTAATCCT-3′) incorporating the Illumina sequencing adapter and a barcode sequence. Then, the amplificated PCR products were purified with Agencourt AMPure XP beads (Beckman Coulter Co., USA). Sequencing was performed on an Illumina NovaSeq6000 with two paired-end read cycles of 250 bases each (Illumina Inc., San Diego, CA). Raw FASTQ files were quality filtered using Trimmomatic (version 0.35) and merged using FLASH (version 1.2.11). Raw sequence reads with a quality score lower than 20 and a length shorter than 50 bases were discarded and clustered into operational taxonomic units (OTUs) with 97% similarity cutoff using Vsearch software (version 2.4.2) and aligned against the Silva database (version 132) for taxonomic classification (Additional file 2: Table S1).

For 16S rRNA gene sequencing analysis, the microbial diversity was calculated using the QIIME tool (version 1.8.0). Differences in alpha diversity were calculated by the Chao1 and Shannon diversity indexes. Beta diversity was determined using QIIME software and visualized in principal coordinates analysis (PCoA) plots. The statistical significance was evaluated with analysis of similarities (ANOSIM). A collinearity diagram was constructed with Circos software to visualize the corresponding abundance relationship between samples and bacterial communities at the family levels. Bacteria with linear discriminant analysis (LDA) score ≥ 3 and P value < 0.05 were defined as the significantly differential expressed bacteria. Function analysis of these representative bacteria was conducted based on Kyoto Encyclopedia of Genes and Genomes (KEGG) and cluster of orthologous groups (COG) annotations.

### Bile salt hydrolase (BSH) activity assay

The BSH activity in the intestinal content was measured based on the generation of CA from TCA. Briefly, 0.05 g content suspended in 1 ml of PBS (pH 7.4) was prepared by ultrasonic and centrifuged to obtain the supernatant and then mixed with TCA. After incubating at 37 °C for 30 min, the reaction was stopped by adding trichloroacetic acid followed by centrifugation. Next, trichloroacetic acid and ninhydrin reagents were added to the supernatant for chromogenic reaction. The O.D. values were detected at an absorbance of 570 nm.

### Statistical analysis

All experimental data were expressed as mean ± SEM and repeated at least three times. One-way ANOVA was employed to compare the differences between multiple groups using GraphPad Prism 8 (Graph-Pad, San Diego, CA). *P* value ≤ 0.05 was considered statistically significant.

See Additional file 1 for the detailed description of other experimental materials and methods.

## Results

### Chemical characterization of SWT

**Fig. 1:**
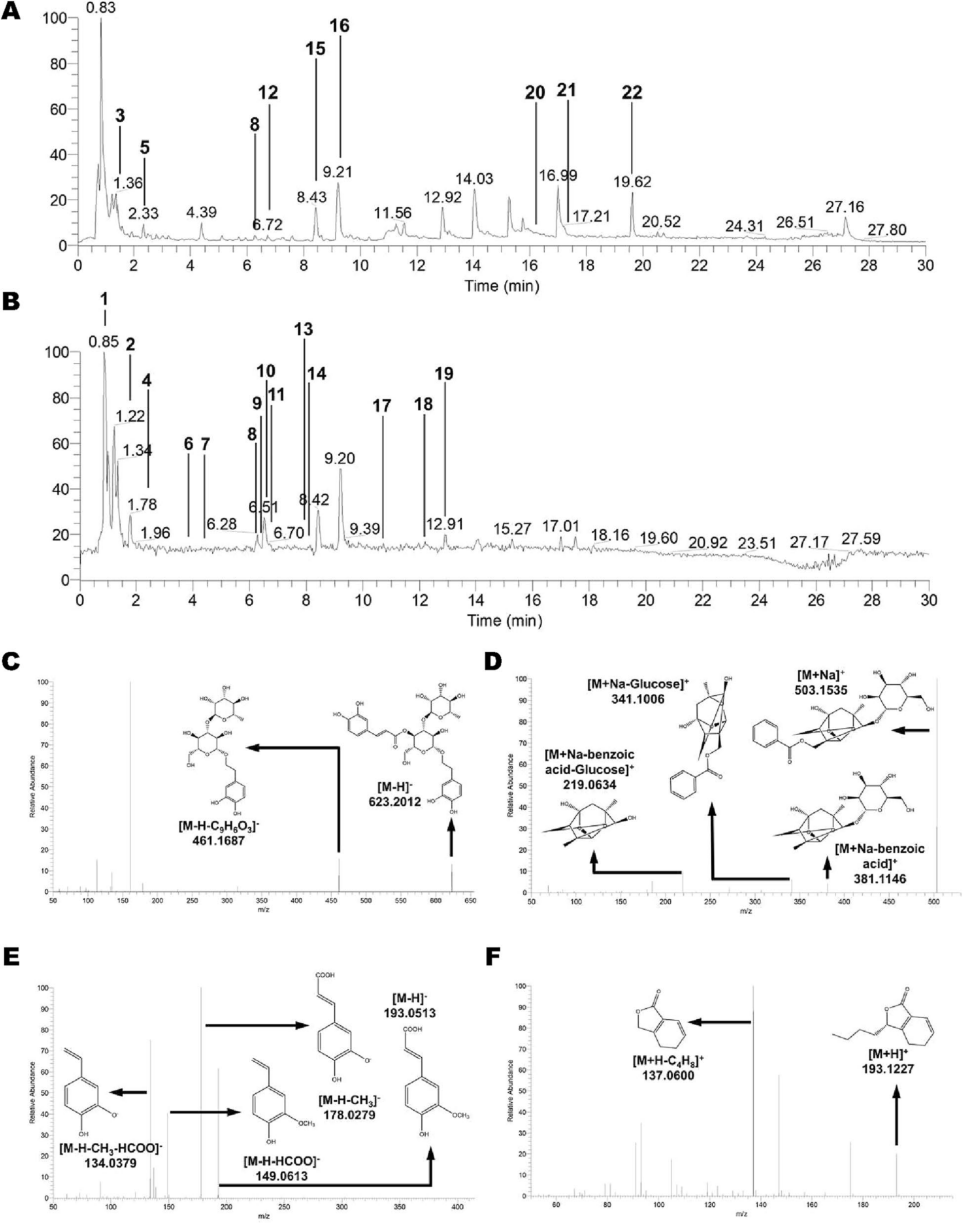
The TICCs of the prepared SWT and the precursor and product ion spectrum of representative compounds. The TICCs of the prepared SWT are obtained in **A** positive (ESI^+^) and **B** negative (ESI^−^) ionization modes. **C** Product ion mass spectrum of deprotonated molecule [M−H]^−^ of verbascoside at m/z 623.2012. **D** Product ion mass spectrum of sodium ionization molecule [M+Na]^+^ of paeoniflorin at m/z 503.1535. **E** Product ion mass spectrum of deprotonated molecule [M−H]^−^ of ferulic acid at m/z 193.0513. **F** Product ion mass spectrum of deprotonated molecule [M+H]^+^ of senkyunolide A at m/z 193.1227

In the current study, a total of 22 representative bioactive compounds derived from RRP, RPA, RAS and RLC were identified in the prepared SWT and were tentatively characterized using the UHPLC coupled with quadrupole-Orbitrap high-resolution mass spectrometry, and the total ion current chromatograms (TICCs) of the positive and negative ionization modes were shown in Fig. 1A, B. Meanwhile, four major ingredients including verbascoside, paeoniflorin, ferulic acid and senkyunolide A have been chosen as the representatives of SWT, and the precursor and production ion spectrum of them were shown in Fig. 1C–F. All the compounds identified from SWT were listed in Table 1 with the detailed information of each ingredient including the exact mass, retention time, precursor and fragment ions in either positive or negative ionization modes. It is worth noting that the polarity was taken into consideration to differentiate the isomers. Moreover, the fragmentation pathways for each compound were described, and the detailed mass spectral and MS/MS data interpretation of all 22 compounds were depicted in Additional file 1.

**Table 1:** Chemical characterization of bioactive compounds in SWT

ID	Compound name	CAS no.	Ret.Time (min)	Positive mode		Negative mode		M.W.
				Measured value	Theoretical value	Measured value	Theoretical value	
1	**Paeonilactone B**	98751-78-1	0.85	n.a	n.a	195.0518 [M−H]^−^	195.0663 [M−H]^−^	196.0736
2	**Citric acid**	77-92-9	1.22	n.a	n.a	191.0204 [M−H]^−^	191.0197 [M−H]^−^	192.0270
3	**Adenosine**	58-61-7	1.39	268.1048 [M+H]^+^	268.104 [M+H]^+^	n.a	n.a	267.0968
4	**Gallic acid**	149-91-7	1.78	n.a	n.a	169.0149 [M−H]^−^	169.0142 [M−H]^−^	170.0215
5	**Phenylalanine**	15099-85-1	2.33	166.0865 [M+H]^+^	166.0863 [M+H]^+^	n.a	n.a	165.0790
6	**Geniposidic acid**	27741-01-1	3.88	n.a	n.a	373.1159 [M−H]^−^	373.1140 [M−H]^−^	374.1213
7	**Catalpol**	2415-24-9	4.32	n.a	n.a	361.1140 [M−H]^−^	361.1135 [M−H]^−^	362.1213
8	**Chlorogenic acid**	327-97-9	6.23	355.0982 [M+H]^+^	355.1204 [M+H]^+^	353.0877 [M−H]^−^	353.0878 [M−H]^−^	394.0951
9	**Oxidized paeoniflorin**	39011-91-1	6.32	n.a	n.a	495.1532 [M−H]^−^	495.1508 [M−H]^−^	496.1581
10	**Caffeic acid**	331-39-5	6.48	n.a	n.a	179.0357 [M−H]^−^	179.0350 [M−H]^−^	180.0423
11	**Vanillic acid**	121-34-6	6.58	n.a	n.a	167.0356 [M−H]^−^	167.0350 [M−H]^−^	168.0423
12	**Xanthotoxin**	298-81-7	6.72	217.0975 [M+H]^+^	217.0495 [M+H]^+^	n.a	n.a	216.0423
13	**RehMapicroside**	104056-82-8	7.89	n.a	n.a	345.1580 [M−H]^−^	345.1555 [M−H]^−^	346.1628
14	**Vanillin**	121-33-5	8.13	n.a	n.a	151.0407 [M−H]^−^	151.0401 [M−H]^−^	152.0473
15	**Albiflorin**	39011-90-0	8.43	503.1535 [M+Na]^+^	503.1524 [M+Na]^+^	n.a	n.a	480.1626
16	**Paeoniflorin**	23180-57-6	9.20	503.1535 [M+Na]^+^	503.1524 [M+Na]^+^	n.a	n.a	480.1626
17	**Ferulic acid**	1135-24-6	9.75	n.a	n.a	193.0513 [M−H]^−^	193.0506 [M−H]^−^	194.0579
18	**Verbascoside**	61276-17-3	12.23	n.a	n.a	623.2012 [M−H]^−^	623.1981 [M−H]^−^	624.2054
19	**Isoacteoside**	61303-13-7	12.94	n.a	n.a	623.2012 [M−H]^−^	623.1981 [M−H]^−^	624.2054
20	**Psoralen**	66-97-7	16.36	187.0757 [M+H]^+^	187.0390 [M+H]^+^	n.a	n.a	186.0317
21	**Dihydrosenkyunolide C**	195142-72-4	17.47	207.1021 [M+H]^+^	207.1016 [M+H]^+^	n.a	n.a	206.0943
22	**Senkyunolide A**	63038-10-8	19.62	193.1227 [M+H]^+^	193.1223 [M+H]^+^	n.a	n.a	192.1150

*n.a.* non applicable; *M.W.* molecular weight

### SWT significantly prevents liver fibrosis in CCl_4_-induced mouse model

**Fig. 2:**
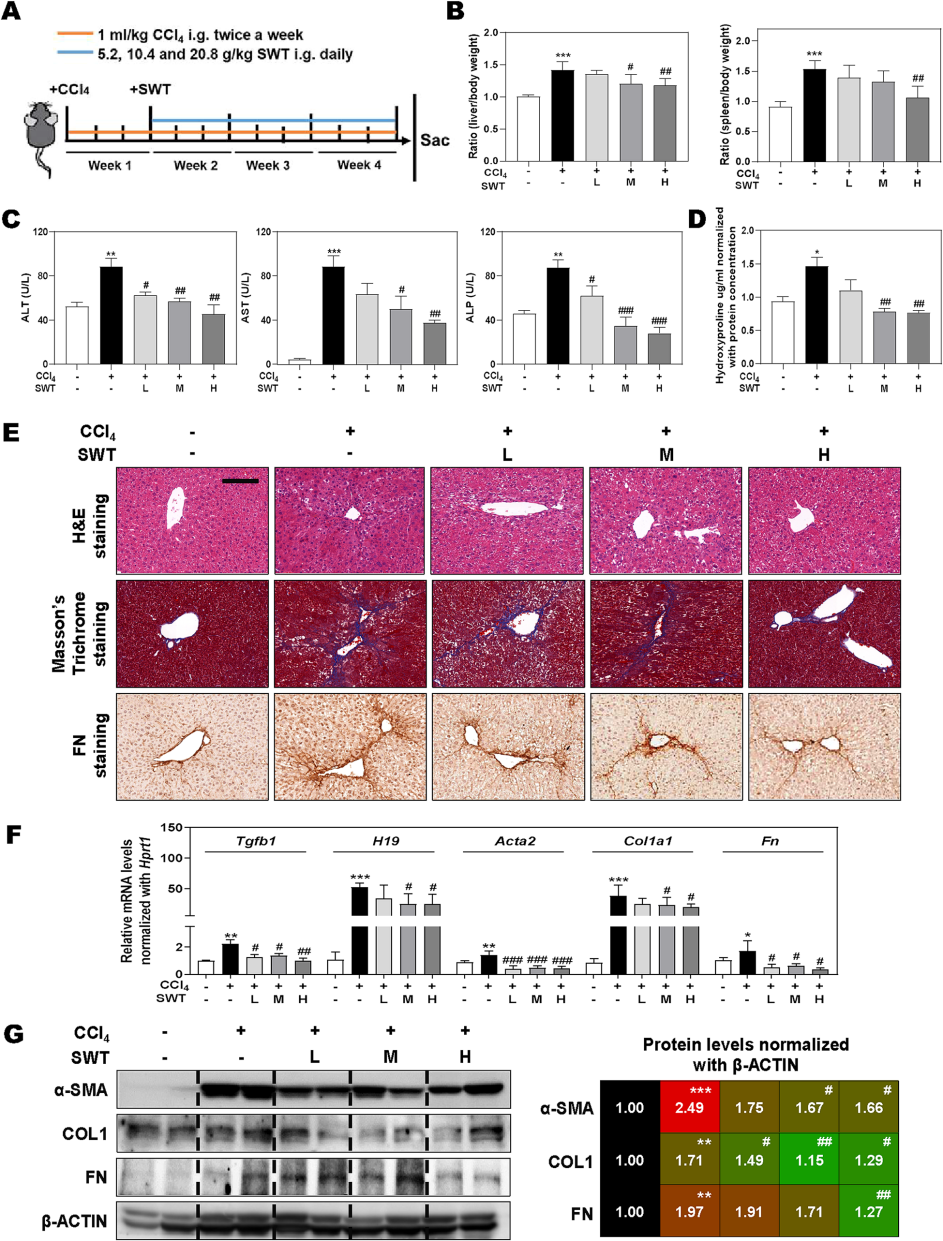
Effects of SWT on CCl_4_-induced fibrotic injury in mice. CCl_4_-induced fibrotic liver injury in mice was established as described in the method. Different doses of SWT were administrated for 3 weeks as indicated. **A** Schematic diagram of in vivo experiments that linked SWT with anti-fibrotic therapies. **B** Ratio of liver and spleen to body weight. **C** ALT, AST and ALP levels in serum. **D** Hepatic hydroxyproline levels. **E** Representative images of H&E, Masson’s Trichrome staining and immunohistochemistry staining against FN of liver tissues. Scale bar = 100 μm. **F** Relative mRNA levels of *Tgfb1*, *H19*, *Acta2*, *Col1a1* and *Fn1* in liver tissues were determined by qPCR and normalized using *Hprt1* as an internal control. **G** Representative immunoblots against α-SMA, COL1, FN and β-ACTIN in liver tissues were shown. Statistical significance: *P < 0.05, **P < 0.01, ***P < 0.001, compared with control group; ^#^*P* < 0.05, ^##^*P* < 0.01, ^###^*P* < 0.001, compared with CCl_4_ group. One-way ANOVA with Tukey’s post-hoc tests (n = 8)

To assess the potential protective effects of SWT on fibrotic liver injury, we administrated different dosages of SWT (5.2, 10.4 and 20.8 g/kg) or vehicle control to C57BL/6J mice with CCl_4_-induced chronic liver fibrosis as described in “Materials and methods” (Fig. 2A). As expected, CCl_4_ gavage markedly induced fibrotic liver injury in mice as indicated by increased liver and spleen indexes, upregulated serum levels of alanine transaminase (ALT), aspartate transaminase (AST) and alkaline phosphatase (ALP) as well as increased hepatic hydroxyproline level compared to that in the control group (Fig. 2B–D). Concurrently, SWT remarkably decreased CCl_4_-induced serum transaminases (Fig. 2C, left and middle panel). Notably, the increased level of ALP and hydroxyproline caused by CCl_4_, biomarkers associated with cholestatic liver injury, were also markedly reversed by SWT administration (Fig. 2C, right panel and Fig. 2D). As shown in Fig. 2E, histopathology examination illustrated by H&E, Masson’s Trichrome staining and immunohistochemistry staining against FN further revealed that CCl_4_ dramatically resulted in fibrotic liver injury accompanied with the increased area of inflammation infiltration, ballooning changes of hepatocytes, increased collagenous fibers and heaptic FN expression, which were all significantly improved by SWT at 10.4 and 20.8 g/kg (Fig. 2E). We previously demonstrated that the aberrant expression of lncRNA H19 has been linked to various fibrotic liver diseases and was upregulated by BAs and oxidative stress [18]. In line with the biochemical and histological findings, transforming growth factor-beta (*Tgfb1*) and *H19* were significantly increased in the livers of mice that underwent CCl_4_ insult but were decreased by SWT (Fig. 2F). Furthermore, the mRNA and protein levels of hepatic markers that could be used to determine the degree of liver fibrosis, such as *Acta2*, *Col1a1* and *Fn1* were markedly elevated by CCl_4_ but inhibited by SWT in different doses (Fig. 2F, G). Collectively, these results suggested that SWT significantly improved CCl_4_-induced cholestatic and fibrotic liver injury.

### SWT ameliorates CCl_4_-induced experimental intestinal barrier dysfunction and hepatic inflammation

**Fig. 3:**
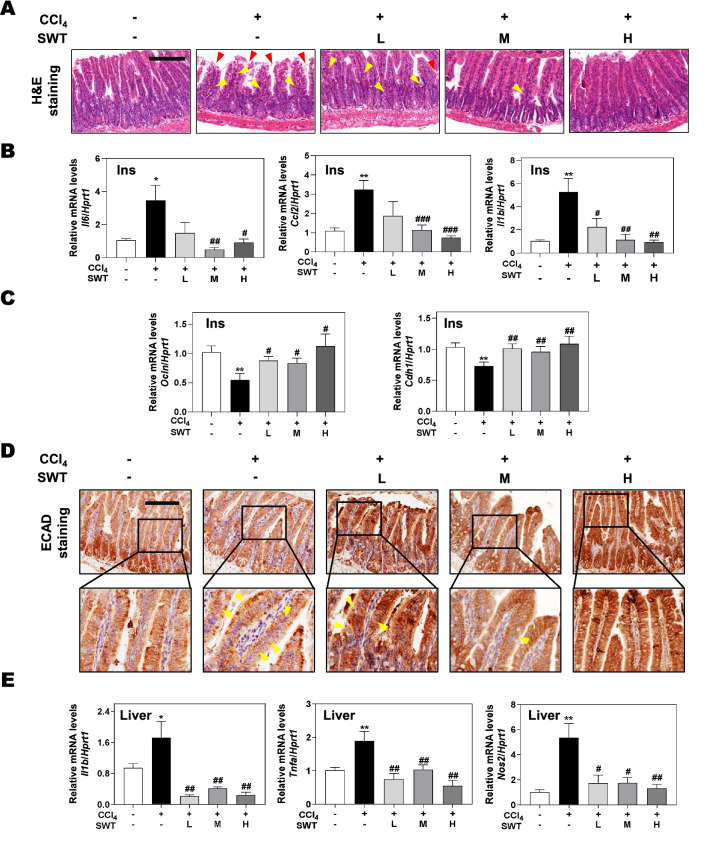
Effects of SWT on intestinal barrier dysfunction and hepatic inflammation in mice with fibrotic liver injury. **A** Representative images of H&E staining of intestine tissues. Scale bar = 100 μm. **B** Relative mRNA levels of *Il6*, *Ccl2* and *Il1b* in intestine tissues were determined by qPCR and normalized using *Hprt1* as an internal control. **C** Relative mRNA levels of *Ocln* and *Cdh1* in intestine tissues were determined by qPCR and normalized using *Hprt1* as an internal control. **D** Representative images of ECAD staining of intestine tissues. Scale bar = 50 μm. **E** Relative mRNA levels of *Il1b*, *Tnfa* and *Nos2* in liver tissues were determined by qPCR and normalized using *Hprt1* as an internal control. Statistical significance: *P < 0.05, **P < 0.01, compared with control group; ^#^*P* < 0.05, ^##^*P* < 0.01, ^###^*P* < 0.001, compared with CCl_4_ group. One-way ANOVA with Tukey’s post-hoc tests (n = 8)

A clinical study suggests that cirrhotic patients manifest intestinal inflammation, altered gut microbiota and increased risk of advanced liver diseases [19]. Coincidentally, increased intestinal mucosal permeability and systemic inflammation were also observed in CCl_4_-treated fibrotic mice due to bacterial translocation from the intestine into the circulation [20]. Considering the pathological correlation between intestine damage and chronic hepatopathy, we thus investigated the effects of SWT on intestinal histopathology in mice with hepatic fibrosis induced by CCl_4_. As shown in H&E staining of the intestine, CCl_4_ significantly induced the infiltration of inflammatory cells (indicated by yellow triangles) and the loss of ileum villi (indicated by red triangles), which were significantly improved by SWT (Fig. 3A). Accordingly, we observed a remarkable increase of the mRNA levels of interleukin 6 (*Il6*), CC-chemokine-ligand-2 (*Ccl2*) and *Il1b* in the CCl_4_ group, indicating the presence of intestinal inflammation in fibrotic mice, which were also significantly reduced by SWT administration (Fig. 3B). On the account of the strong correlation between increased inflammatory cytokines and destructed intestinal barrier, we next determined the gene expression of tight junction proteins and explored whether the destructed intestinal mucosal barrier was improved by SWT. As shown in Fig. 3C, the mRNA levels of Occludin (*Ocln*) and ECAD (*Cdh1*), genes encoding typical tight junction proteins were all decreased in the CCl_4_ group, which were then significantly increased by SWT at different doses. Consistently, immunohistochemistry staining against ECAD further revealed that the SWT improved CCl_4_-induced loss and mislocalization of ECAD in the intestinal epithelium (indicated by yellow triangles), implying that the destruction of intestinal mucosal barrier caused by CCl_4_ was revered by SWT administration (Fig. 3D). Considering the pathophysiological role of the gut-liver axis in the liver fibrosis progression, we next measured the mRNA levels of pro-inflammatory factors in the liver. As shown in Fig. 3E and Additional file 1: Fig. S1, SWT in different doses all remarkably decreased the mRNA levels of *Il1b*, tumor necrosis factor-alpha (*Tnfa*), inducible nitric oxide synthase (iNOS/*Nos2*), *Ccl2* and its receptor *Ccr2*, when compared to the CCl_4_ group. These results suggested that SWT alleviated CCl_4_-induced hepatic inflammation and liver fibrosis through mechanisms not only the prevention of intestinal inflammatory response but also the regulation of hepatoenteral interaction.

### SWT restructures the intestinal microbiota in fibrotic mice

**Fig. 4:**
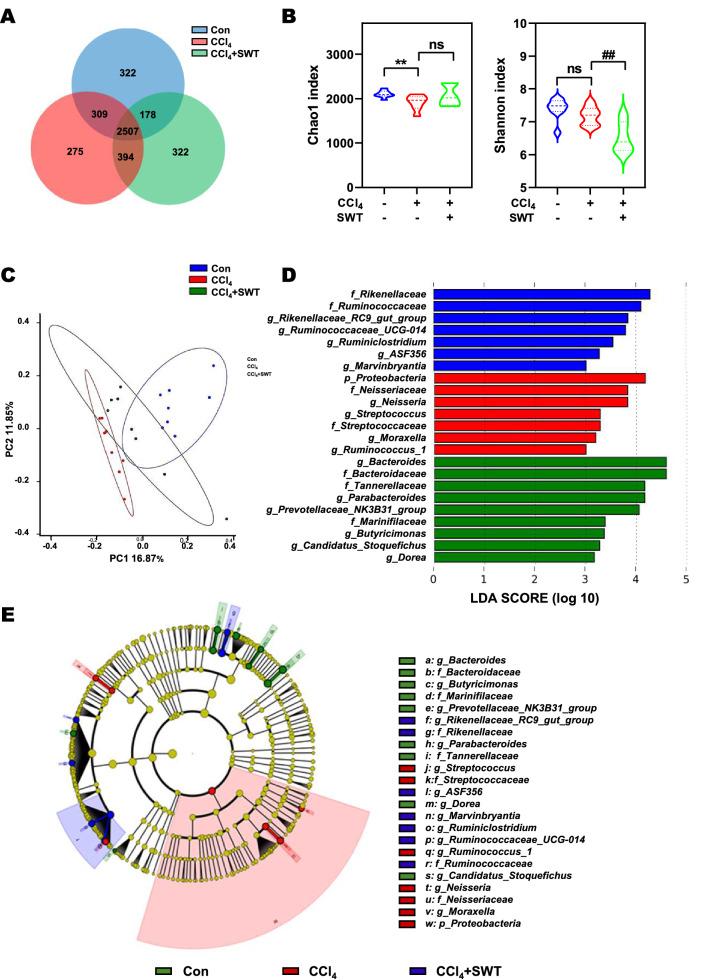
Effects of SWT on the diversity and composition of intestinal microbiota in mice with fibrotic liver injury. **A** The Venn diagram analysis of OTUs. **B** Alpha diversity was evaluated based on the Chao1 (left panel) and Shannon (right panel) indices of the OTU levels. **C** The dissimilarities in microbial composition on OTU level was determined by PCoA. Each dot represented an individual sample. **D**, **E** LEfSe analysis was used to identify the most differentially abundant taxa of bacterial communities from phylum to genus level (LDA score ≥ 3.0)

To comprehensively reveal the mechanism underlying the therapeutic effects of SWT on the injured liver and intestine, the bacterial DNA in intestinal contents was extracted and analyzed by 16S rRNA-sequencing analysis. Of note, SWT exposure markedly altered the diversity of intestinal microbiota in mice. As shown in the Venn’s diagram analysis of OTUs (Fig. 4A), 2507 uniform OTUs were identified in three groups based on 97% sequence similarity. Compared to the control group, the specific OTUs (275) in the CCl_4_ group were decreased but increased after SWT treatment (322). Both α and β diversity analyses were further performed to evaluate the differences in community diversity of intestinal bacteria among various groups. The community abundance (Chao1) and diversity index (Shannon) were used to reflect the α diversity among groups. Compared with the control group, CCl_4_ exposure markedly decreased the Chao1 index, which was then slightly increased by SWT administration (Fig. 4B, left panel). Although there was no clear separation of diversity between the control group and the CCl_4_ group, a markedly distinct difference between the CCl_4_ group and the CCl_4_ + SWT group was observed (Fig. 4B, right panel). In addition, a PCoA analysis was performed to analyze the β diversity and showed that the intestinal microbiota distributions among three groups were separately clustered with different groups (Fig. 4C). We further analyzed the variation of gut microbiota at different taxonomic ranks based on the results of 16S rRNA gene sequencing. LDA coupled with effect size (LEfSe) analysis was utilized to analyze the gut microbiota composition in different groups and established 23 bacterial clades performing biologically and statistically consistency of differences from phylum to genus levels (Fig. 4D, E). It was worth noting that bacteria with high BSH activity were the dominant genuses and families in CCl_4_ + SWT group, such as *Bacteroidetes*, *Bacteroidaceae* and *Prevotellace_NK3B31_group*, while the dominant bacteria in the CCl_4_ group were *Proteobacteria*, *Streptococcaceae* and *Streptococcus*. Taken together, our results demonstrated that SWT treatment strikingly restructured gut microbiota in fibrotic mice.

### SWT improves CCl_4_-mediated disturbance of gut microbiota and their associated biological functions

**Fig. 5:**
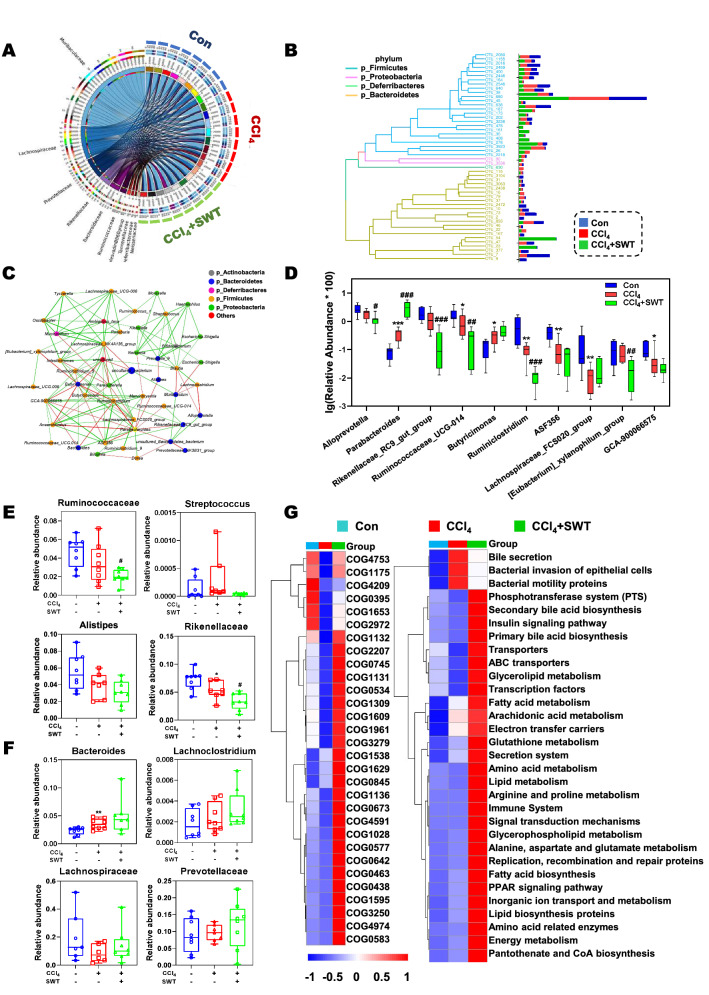
Effects of SWT on CCl_4_-mediated disturbance of gut microbiota and their associated biological functions. **A** The correlations between microbiota and samples were detected using Circos analysis. **B** Top 50 OTUs level classifiers for different groups were shown with phylogenetic tree. **C** Correlation network analysis of the 44 most abundant genera (*P* < 0.01). The lines between nodes indicated the Spearman correlation, and the color intensity indicated the correlation coefficient (red, positive; green, negative). Genera color were based on phylum affiliation, and sizes indicated mean relative abundance. **D** The relative abundance (%) of the top 10 bacteria (genus). **E** Relative abundance of pathogens, including *Ruminococcaceae*, *Streptococcus*, *Alistipes* and *Rikenellaceae*. **F** Relative abundance of probiotics, including *Bacteroides*, *Lachnoclostridium*, *Lachnospiraceae* and *Prevotellaceae*. **G** The analysis of COGs (left panel) and KEGG (right panel) pathways in Con, CCl_4_ and CCl_4_ + SWT groups. Statistical significance: *P < 0.05, **P < 0.01, ***P < 0.001, compared with control group; ^#^*P* < 0.05, ^##^*P* < 0.01, ^###^*P* < 0.001, compared with CCl_4_ group. One-way ANOVA with Tukey’s post-hoc tests (n = 8)

To visualize the intermicrobial associations among control, CCl_4_ and CCl_4_ + SWT groups, circos analysis about top ten families was carried out (Fig. 5A). In addition, top 50 OTUs level classifiers for different groups were shown with a phylogenetic tree to display the evolutionary relationships among intestinal bacteria at the phylum level (Fig. 5B). Moreover, network correlation analysis was performed to reveal the relationships among intestinal microbial communities and shown in Fig. 5C. The green line indicated a positive correlation and the red line presented a negative correlation between each species at the genus level. The size of nodes and edges represented the abundances of microbial community and degree of relevance of different species, respectively. At the genus level, the differences in the composition of microbiota among different groups were shown in Fig. 5D; the relative abundance of *Parabactertoides* was increased but *Rikenellaceae_RC9_gut_group* and *Ruminococcaceae_UCG-014* were significantly decreased by the administration of SWT. Specifically, several pernicious bacteria, especially for *Ruminococcaceae*, *Streptococcus*, *Alistipes* and *Rikenellaceae* were decreased (Fig. 5E and Additional file 1: Fig. S2A), while several probiotics, especially for *Bacteroides*, *Lachnoclostridium*, *Lachnospiraceae* and *Prevotellaceae* were significantly increased in CCl_4_ + SWT group, compared to that in CCl_4_ group (Fig. 5F and Additional file 1: Fig. S2B). Except for microbial profiling, we performed PICRUSt to predict bacterial function and demonstrated that SWT strikingly reshaped gut microbiota and further led to changes in the functional composition of microbial communities. Of note, clusters of orthologous groups (COGs) analysis revealed that the functional abundances of COG1131, COG1653, COG0395 and COG4591 (ABC-type multidrug transport system) were all inhibited in response to CCl_4_ challenge, which were significantly increased by SWT treatment (Fig. 5G, left panel). In the KEGG analysis (Fig. 5G, right panel), a knowledge base for systematic analysis of gene functions, several pathways related to lipid metabolism, ABC transporters and immune system were upregulated after SWT administration. Significantly, BA-related pathways, such as bile secretion, primary and secondary BA biosynthesis pathways, were also altered in CCl_4_ + SWT group, suggesting the plausible bioactivities of SWT in regulating BA homeostasis in the intestinal microbiota of fibrotic mice. Collectively, SWT improved the disturbance of intestinal microbiota and their associated biological functions, which might be one of the vital mechanisms for alleviating fibrotic liver injury via liver-gut axis.

### SWT alters BA-biotransforming bacteria and serum BAs profiling

**Fig. 6:**
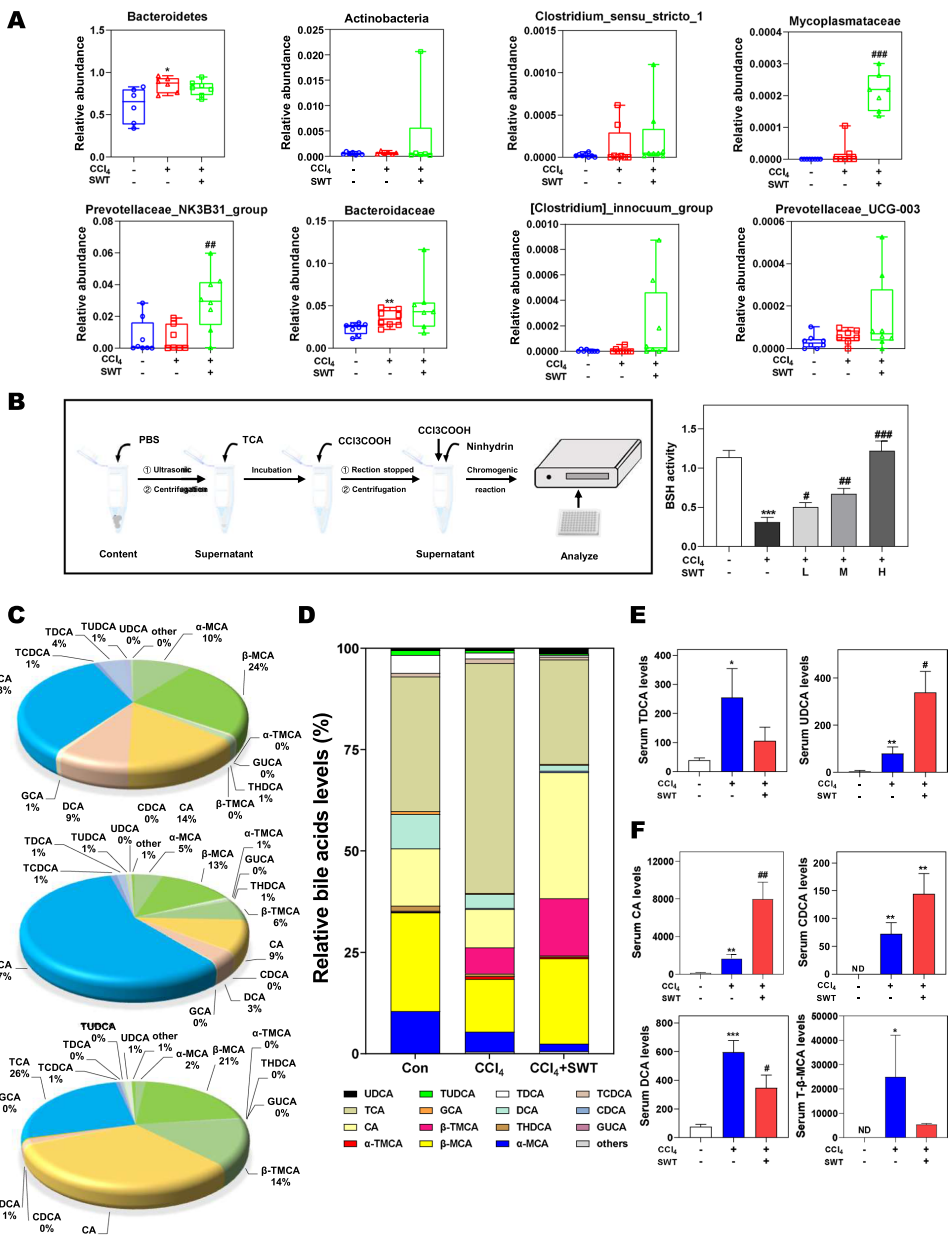
Effects of SWT on BA-biotransforming bacteria and serum BAs profiling. **A** Relative abundance of *Bacteroidetes*, *Actinobacteria*, *Clostridium_sensu_stricto_1*, *Mycoplasmataceae*, *Prevotellaceae_NK3B31_group*, *Bacteroidaceae*, *[Clostridium]_innocuum_group* and *Prevotellaceae_UCG-003*. **B** The process flow diagram of BSH activity assay (left panel) and the level of BSH in intestinal contents (right panel). **C** Changes in bile acid composition. **D** The relative abundance (%) of the top 16 BAs. The serum levels of **E** TDCA, UDCA, **F** CA, CDCA, DCA and T-β-MCA. Statistical significance: *P < 0.05, **P < 0.01, ***P < 0.001, compared with control group; ^#^*P* < 0.05, ^##^*P* < 0.01, ^###^*P* < 0.001, compared with CCl_4_ group. One-way ANOVA with Tukey’s post-hoc tests (n = 8)

Another critical function carried out by the gut microbiota is the deconjugation of BAs by BSH enzymes, which facilitates the reabsorption of unconjugated BAs and promotes the production of secondary BAs. Interestingly, SWT treatment markedly increased the BSH activity of *Bacteroidetes*, *Actinobacteria*, *Clostridium_sensu_stricto_1*, *Mycoplasmatace*, *Prevotellaceae_NK3B31_group*, *Bacteroidaceae*, *[Clostridium]_innocuum_group* and *Prevotellaceae_UCG-003* from phylum to genus, compared to the control and CCl_4_ groups (Fig. 6A). To further verify whether BSH activity was increased after SWT administration, we directly determined the BSH activities in intestinal content using an enzymatic assay described in “Materials and methods” (Fig. 6B, left panel). As shown in Fig. 6B, right panel, the BSH activity of intestinal content was significantly downregulated in the CCl_4_ group but further upregulated in a dose-dependent manner after SWT administration, suggesting that SWT might regulate BA metabolism by promoting BA deconjugation. It has been previously reported that abnormal BA accumulation or disruption of intrahepatic BA circulation changed the composition of microbiota and influenced intestinal inflammation [21]. To further investigate the effects of SWT on the composition of serum BAs, we measured more than 45 different BAs in the serum using a high accuracy LC–MS/MS assay. As shown in Fig. 6C, D, CCl_4_ gavage significantly changed the ratios of primary BAs to secondary BAs and unconjugated BAs to CBAs. The most obvious changes in the CCl_4_ group were the dramatic increase of TCA level and decrease of CA level in the serum, compared to that in the control group (Fig. 6D). Interestingly, CCl_4_-induced changes of TCA and CA were markedly reversed by SWT. Several studies reported that taurodeoxycholic acid (TDCA) functioned as a cytotoxic BA to promote liver fibrosis in mice, while UDCA, a beneficial BA, can eliminate the hepatic accumulation of toxic BAs [21]. Notably, SWT not only suppressed the increase of TDCA but also strengthened the compensatory increase of UDCA caused by CCl_4_ (Fig. 6E). It is noteworthy that in Fig. 6F, upper panel, BAs like CA and CDCA, which acted as FXR ligands to regulate different signaling involved in hepatocyte repair, ductular reaction and HSC deactivation, were significantly increased after SWT administration compared to the CCl_4_ group. Meanwhile, naturally occurring FXR antagonists like DCA and tauro-β-muricholic acid (T-β-MCA) were decreased after SWT treatment (Fig. 6F, down panel). These results, combined with the effects of SWT treatment on intestinal bacteria, provided compelling evidence that SWT might influence the bidirectional interplay between BAs and gut microbiome.

### SWT modulates BA transport and synthesis through the activation of FXR and its downstream signaling

**Fig. 7:**
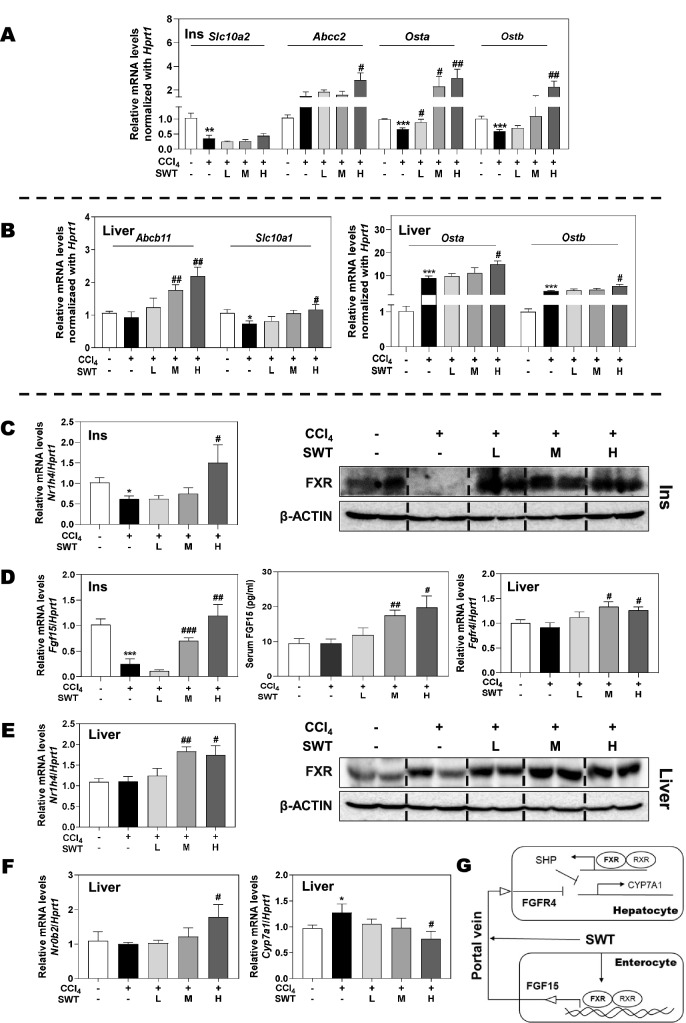
Effects of SWT on FXR and its downstream signaling in mice with fibrotic liver injury. Relative mRNA levels of **A** *Slc10a2*, *Abcc2*, *Osta*, *Ostb*, **C** *Nr1h4* (left panel) and **D** *Fgf15* (left panel) in the intestine, **B** *Abcb11*, *Slc10a1*, *Osta*, *Ostb*, **D** *Fgfr4* (right panel), **E** *Nr1h4* (left panel), **F** *Nr0b2* and *Cyp7a1* in the liver were determined by qPCR and normalized using *Hprt1* as an internal control. Representative immunoblots against FXR and β-ACTIN in the intestine (**C**, right panel) and liver (**E**, right panel) were shown. **D** Serum levels of FGF15 were determined by FGF15 ELISA assay kit (middle panel). **G** The role of SWT in regulating “liver-gut axis” involved pathways. Statistical significance: *P < 0.05, **P < 0.01, ***P < 0.001, compared with control group; ^#^*P* < 0.05, ^##^*P* < 0.01, ^###^*P* < 0.001, compared with CCl_4_ group. One-way ANOVA with Tukey’s post-hoc tests (n = 8)

To delineate the underlying mechanisms via which SWT regulated BA metabolism and prevented chronic liver fibrosis progression, we next measured the mRNA expression of different BA transporters associated with FXR signaling in intestine and liver, respectively. As shown in Fig. 7A, the intestinal mRNA expression of apical sodium-dependent bile acid transporter (ASBT, *Slc10a2*), a BA transporter responsible for reabsorbing CBAs into the liver, was suppressed in both CCl_4_ and CCl_4_ + SWT groups. Multidrug resistance-associated protein 2 (MRP2/*Abcc2*), MRP3 (*Abcc3*) and organic solute transporter alpha/beta (OSTα/β) are responsible for transporting BAs to serum. The mRNA levels of *Abcc2* and *OSTa/b* in intestinal epithelial cells were unchanged or slightly decreased in the CCl_4_ group compared with that in the control group but significantly increased almost two- to fourfolds by SWT at high dose. However, the mRNA level of *Abcc3* in the intestine was not changed either in CCl_4_ or CCl_4_ + SWT groups (Additional file 1: Fig. S3A). Furthermore, we examined the relative mRNA levels of genes involved in BA uptake and efflux in the liver. Our qPCR results revealed that the mRNA levels of bile salt export pump (BSEP, *Abcb11*), a BA transporter responsible for BA excretion into bile canaliculi, and sodium taurocholate co-transporting polypeptide (NTCP, *Slc10a1*), a BA transporter responsible for reabsorbing BAs into the liver, were all significantly increased by SWT administration (Fig. 7B, left panel). Although SWT didn’t alter *Abcc2* and *Abcc3* mRNA levels (Additional file 1: Fig. S3B), it markedly resulted in the enhanced transcription of *OSTa/b* in the liver when compared to the control and CCl_4_ groups (Fig. 7B, right panel), thereby confirming the role of SWT in promoting hepatic BA efflux and inhibiting BA uptake. On account of the critical role of FXR played in the transport and circulation of BAs, we then investigated the intestinal expression of FXR and demonstrated that CCl_4_ dramatically decreased the mRNA and protein expressions of FXR (*Nr1h4*) in the ileum, which were markedly increased by SWT (Fig. 7C and Additional file 1: Fig. S4A). Besides its local effects in the liver, the activation of FXR in ileal enterocytes promotes the expression of FGF15, which subsequently acts on the FGFR4/b-klotho heterodimer receptor complex to suppress BA synthesis [7]. Thus, we wondered whether the SWT-induced activation of intestinal FXR may be critical to its beneficial effect on livers. The qPCR and ELISA data further showed that in this CCl_4_-caused fibrotic mouse model, the ileum and serum expression of *Fgf15* and hepatic mRNA level of *Fgfr4* were decreased or almost unchanged, which were all significantly increased by SWT treatment (Fig. 7D). Although the hepatic FXR expression remained unchanged in response to CCl_4_ insult, SWT not only remarkably increased the mRNA level of *Nr1h4* but also its protein level in the liver (Fig. 7E and Additional file 1: Fig. S4B). Ileal FXR activation contributes to the strong inhibition of CYP7A1, whereas hepatic FXR activation also regulates CYP7A1-mediated BA synthesis due to the induction of SHP (*Nr0b2*). As expected, SWT at a high dose significantly induced *Nr0b2* transcription and decreased *Cyp7a1* expression, respectively (Fig. 7F). These findings suggested that SWT regulated BA transportation and repressed CYP7A1 transcription by activating FXR-FGF15 enterohepatic and FXR-SHP hepatic pathways (Fig. 7G).

## Discussion

**Fig. 8:**
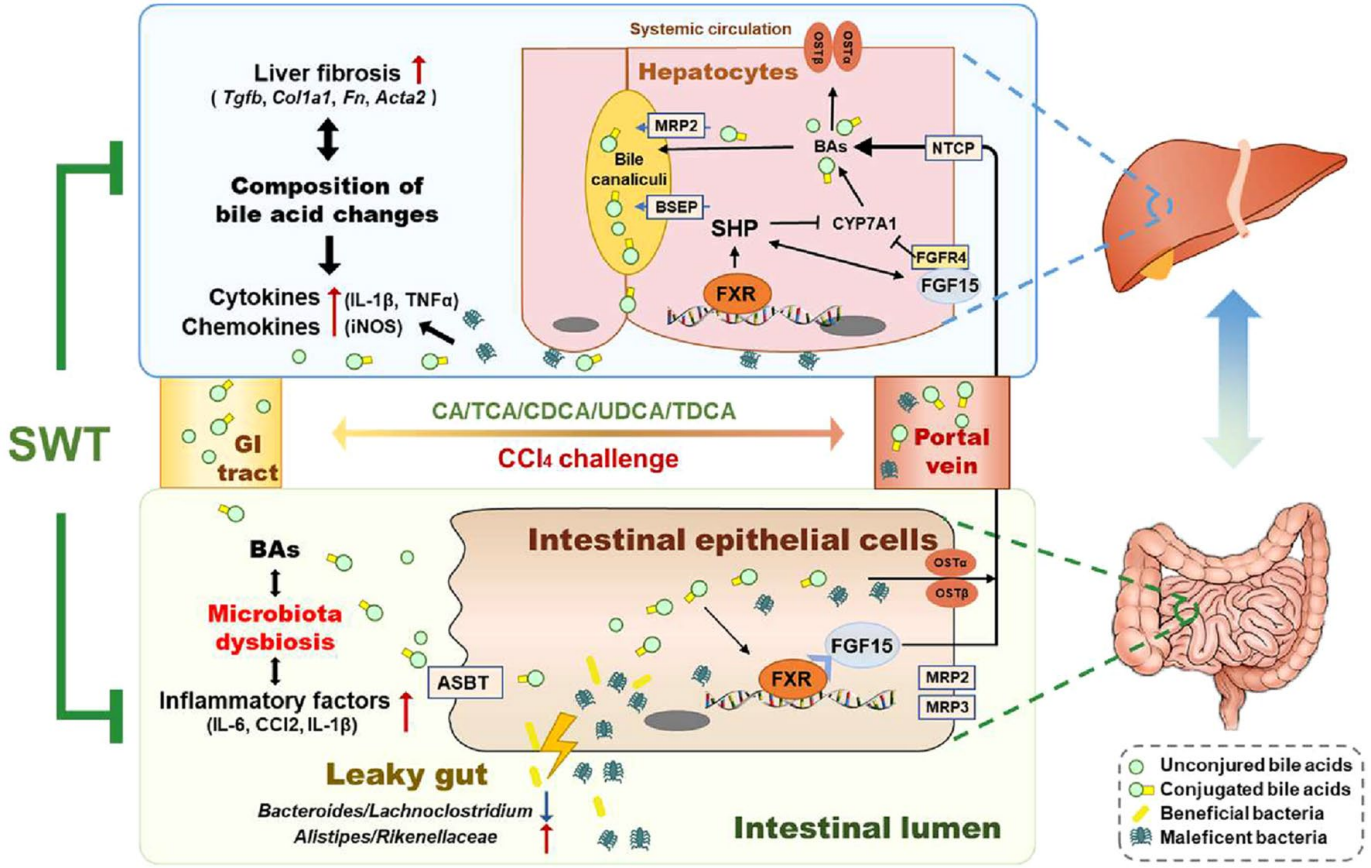
Schematic diagram of the proposed mechanisms underlying the protective effects of SWT on fibrotic liver injury

SWT, a TCM formula used in clinical practice to treat menstrual discomfort and infertility for centuries, has recently been investigated for its therapeutic effects on chronic liver diseases [22]. However, the underlying mechanisms of SWT against fibrotic liver injury remain to be further elucidated. Here, our data demonstrated that SWT had a remarkable therapeutic effect on CCl_4_-induced liver fibrosis, as evidenced by significantly decreased extracellular matrix deposition in livers, intestinal barrier dysfunction as well as hepatic and intestinal inflammatory response. Next by performing 16S rRNA sequencing and UHPLC-MS/MS to probe the effect of SWT on the intestinal microbiota and BA homeostasis of mice with fibrotic liver injury, we demonstrated that the beneficial effects of SWT may be dependent on restructuring gut microbiota including the changes of microbiota composition and relative abundance and the increase of bacteria with BSH activities. Notably, after SWT administration, unconjugated BAs like CA and CDCA in serum were dramatically increased while CBAs including TCA and TDCA were decreased, indicating that SWT might alter the composition of BAs in circulation by regulating gut microbiota. Our results further elucidated that SWT effectively depressed BA de novo synthesis and enhanced BA excretion mediated by FXR-FGF15 enterohepatic and FXR-SHP hepatic pathways, which also modulated enterohepatic BA circulation and relieving liver injury (Fig. 8). These results highlighted the potential roles of BA-related gut-liver axis played in the therapeutic effects of SWT.

In the clinical setting, chronic liver diseases are frequently associated with GI disorders through enterohepatic circulation. About 7.5% of colitis patients typically present with fibrotic cholangitis characterized by BA over-accumulation, while more than 60% of patients with fibrotic cholangitis often co-occur with inflammatory bowel diseases [23]. This connection between fibrotic liver disease and recurrent intestinal injury may result from systemic inflammation and the disruption of the intestinal barriers of defense caused by microbiota translocation and PAMPs-associated immune hyperactivation [24]. Collectively, intense research over recent years has emphasized the importance of targeting the liver-gut axis for the treatment of hepatopathies, which is also in accordance with the theory and therapeutic viewpoint of TCM. Here, we reported that SWT dramatically alleviated both fibrotic liver injury and intestinal barrier damage caused by CCl_4_ (Figs. 2 and 3), providing a novel treatment for hepatopathies targeting the liver-gut axis, which might be involved in the changes of intestinal microbiota and BA metabolism.

The translocation of bacteria and bacterial endotoxins like lipopolysaccharide (LPS) and DNA/RNA fragments into systemic circulation combined with activated macrophages may be the primary reason for eliciting enterohepatic inflammation and pro-fibrotic hepatobiliary responses [25]. Prior studies suggest that many pathogenic bacteria producing LPS are positively correlated with hepatic and intestinal inflammation. It was reported that the abundance of facultative pathogenic *Alistipes* was positively correlated with serum LPS level and the colonization by *Alistipes* in IL10^−/−^ mice induced colitis and colonic tumors by activating the IL-6-STAT3 axis [26]. Additionally, *Ruminococcaceae* and *Veillonellaceae* were recorded as the most representative bacterial taxa associated with altered BA composition and the severity of liver fibrosis [27]. Nevertheless, except for detrimental microbiota, probiotics offer great potential benefits and can be used to treat inflammatory disorders in the digestive tract through themselves or their secreted products. Carlo Selmi et al. [28] reported the decreased gut probiotics including *Faecalibacterium* and *Bacteroides* in a small cohort of patients with biliary cholangitis. A correlation analysis indicated that *Lachnospiraceae* and other probiotics were negatively associated with the production of several pro-inflammatory cytokines and further alleviated colon inflammation [29]. Consistently, we found that SWT directly decreased the abundances of *Ruminococcaceae*, *Alistipes*, *Veillonellaceae* and *Rikenellaceae* and elevated the inhibition of *Bacteroides* and *Lachnospiraceae* caused by CCl_4_ (Figs. 4, 5 and Additional file 1: Fig. S2), which may be one of the main mechanisms for SWT alleviating inflammation in the liver and gut.

It is worth noteworthy that BA is another one of the main causes of enterohepatic inflammation, which can be interacted with microbiota bidirectionally. On the one hand, emerging evidence indicates that BAs change the environment of gut microbiota by inhibiting the growth of susceptible bacteria and promote fibrotic liver injury. We previously demonstrated that increased BAs induced loss of stemness in intestinal stem cells, disruption of intestinal barrier function, bacterial translocation, activation of hepatic inflammation and liver fibrosis [30]. On the other hand, microbiota enriched in the intestine is also involved in BA metabolism. Most of the CBAs like TCA are significantly hydrolyzed to free BAs by gut microbiota with BSH activity. Previous and our recent studies also showed that TCA induced the hepatic expression of H19 and fibrotic markers, triggered HSC activation and promoted systemic inflammation and liver fibrosis [10, 31]. It is worth noting that the serum level of TCA was markedly decreased but the serum level of CA was significantly increased after SWT treatment, accompanied by increased BSH activity in the intestinal content (Fig. 6). It seems likely that the metabolism of TCA into CA is increased due to BA-biotransforming bacteria after SWT treatment. From another perspective, BA transporters that regulate the BA enterohepatic circulation also exert different BA affinities for different BAs and influence the transport and metabolism of BAs. OSTα/β, localized in the basolateral membrane for transporting BAs to serum, has a stronger affinity for unconjugated BAs than CBAs [32]. Nevertheless, BSEP and MRP2, which mediate the transport of BAs from hepatocytes into bile canaliculi, and ASBT, expressed at the apical membrane of intestinal epithelial cells, all preferentially transports CBA compared with unconjugated BAs [33]. Here, we not only confirmed the persistent inhibitory effects of SWT on ileum ASBT level but also found that SWT more efficiently enhanced the expression of OSTα/β, BSEP and MRP2 in the liver and intestine (Fig. 7), which provided another explanation for the increased ratio of unconjugated BAs to CBAs. However, a systems biology perspective is still required to elucidate the exact way that SWT regulates the liver-gut-BAs-microbiota axis in the fibrotic mouse model.

The feedback inhibition of CYP7A1 by BAs is mediated either by the enterohepatic FXR-FGF15 axis or a nuclear receptor signaling cascade involving FXR and SHP. Studies so far have attributed the hepatoprotective effects of intestine-derived FGF15 largely to the activation of gut-liver signaling feedback inhibition of CYP7A1, and the activation of FGF15 has been thought to provide promise for improving liver disease pathogenesis. Notably, Kim et al. [34] observed increased levels of T-α/β-MCA after FGF19 injection in wild-type mice but not in SHP-knockout mice, suggesting that SHP is required for FGF15/19 to efficiently regulate BA synthesis. Although the basal expression of FGF15 in the intestine is low while the basal SHP expression in the liver is relatively high, prior studies have suggested that induction of intestinal FGF15 by FXR is more essential for short-term repression of CYP7A1 than the induction of hepatic SHP by FXR. There is now definitive evidence that FXR-selective agonist GW4064 significantly suppressed CYP7A1 in liver-specific but not in intestine-specific FXR-null models [7]. In this study, we identified that SWT not only upregulated intestinal FXR expression and induced the serum and ileum levels of FGF15, but also activated the hepatic FXR-SHP pathway to cooperatively inhibit BA synthesis, suggesting a link between FGF15-SHP axis and SWT-mediated BA homeostasis. In addition, a previous study reported that T-β-MCA competitively inhibited the activation of FXR by other BAs and the reduced level of T-β-MCA contributed to the increased expression of FGF15 in the ileum through FXR activation [35]. Consistently, we found that SWT markedly decreased the serum T-β-MCA level compared to that in the CCl_4_ group (Fig. 6), which improved the feedback regulation of BA metabolism and reduced the risk of fibrotic liver injury. A previous study reported that an FXR-agonist OCA and fexaramine were reported to decrease the translocation of GFP-*Escherichia coli* from gut to liver and improve liver injury in CCl_4_-induced cirrhotic mice [36]. We speculate that SWT shares a similar mechanism of action with other reported FXR agonists (Fig. 7). However, whether SWT contains the potential FXR agonists or activates FXR signals by regulating BA metabolism is not investigated in the present work, and further studies are still required.

To further develop novel SWT-based therapies, it is necessary to identify representative bioactive components in SWT contributing to the pharmacological anti-hepatic fibrosis effects, especially regulatory effects on the liver-gut axis. In the current study, we preliminarily identified 22 most abundant bioactive monomers in SWT, especially for paeoniflorin from RPA, ferulic acid from RAS, verbascoside from RRP and senkyunolide A from RLC. Emerging evidence shows that several drugs with the potential to enhance the expression or rescue the function of BA transporters are being evaluated in clinical trials for treating fibrotic liver diseases, like several ASBT inhibitors [37] or UDCA, 4-PB and Ivacaftor [38]. In the current study, we preliminarily demonstrated that SWT was implicated in the regulation of BA transporters. A previous study reported that paeoniflorin remarkably restored the expression of BSEP, MRP2 and NTCP but not inhibited BA synthesis in a cholestatic mouse model [39]. In addition, paeoniflorin was reported to ameliorate experimental fibrotic liver injury and colitis by decreasing ECM accumulation, inhibiting whole-body inflammatory responses, reshaping the gut microbiome community and repairing intestinal barrier damage [40, 41], suggesting that the anti-hepatic fibrosis effects of SWT are at least partially depending on paeoniflorin. Ferulic acid has been acknowledged to be another effective component isolated from SWT with the potential for ameliorating liver fibrosis by inhibiting the TGF-β/Smad pathway and altering the composition of gut microbiota, for example, increasing the proportion of *Bacteroidetes* [42, 43]. However, the hepatoprotective effects of verbascoside and senkyunolide A are almost neglected, and only a few studies briefly evaluated their immunomodulatory effects without investigating underlying mechanisms. Future studies defining the mechanisms underlying the beneficial effects of monomers or a combination of ingredients in SWT on fibrotic liver injury with BA and microbiota imbalance signatures will be of great basic research and translational value.

## Conclusion

In summary, our data revealed that SWT improved pathogenic BA profile by modulating gut microbiota, which then altered BA profile and bacteria-related intestinal and hepatic inflammation, and eventually alleviated liver fibrosis. Our study strongly suggests that SWT and its bioactive ingredients represent a promising therapeutic strategy in the treatment of fibrotic liver injury during clinical application.

## Supplementary Information

**Additional file 1.** Materials and methods for other experiments and additional figures.

**Additional file 2: Table S1.** Primer sequences used in qPCR.

## Data Availability

All data included in this article are available from the corresponding author upon request.

## Abbreviations

ASBT: Apical sodium-dependent bile acid transporter
BA: Bile acid
BSEP: Bile salt export pump
BSH: Bile salt hydrolase
CA: Cholic acid
CBA: Conjugated bile acid
CCl_4_: Carbon tetrachloride
CYP7A1: Cholesterol 7α-hydroxylase
FGF15: Fibroblast growth factor 15
FXR: Farnesoid X receptor
MRP2: Multidrug resistance-associated protein 2
OSTα/β: Organic solute transporter alpha/beta
SHP: Small heterodimer partner
SWT: Si-Wu-Tang
TCA: Taurocholic acid
