# Supplementary material for: Si-Wu-Tang ameliorates fibrotic liver injury via modulating intestinal microbiota and bile acid homeostasis
Source: Chin Med. 2021 Nov 4;16:112. doi: 10.1186/s13020-021-00524-0 (PMC8570021; doi:10.1186/s13020-021-00524-0)
Supplement: Supplementary file 1 — Additional file 1. Materials and methods for other experiments and additional figures. [file 13020_2021_524_MOESM1_ESM.docx]

**Materials and methods for other experiments and additional figures**

**1. Additional materials and methods**

**1.1 Chemical Characterization of SWT**

The molecular formula of paeonilactone B **(1)** is C_10_H_12_O_4_. Paeonilactone B exhibited a deprotonated molecule [M-H]^−^ at m/z 195.0578 in negative ionization mode. The deprotonated molecule lost a H_2_O, forming a fragment ion [M-H−H_2_O]^-^ at m/z 177.0410.

The molecular formula of citric acid **(2)** is C_6_H_8_O_7_. Citric acid exhibited a deprotonated molecule [M–H]^-^ at m/z 191.0204 in negative ionization mode. The deprotonated molecule lost a H_2_O, forming a fragment ion [M-H−H_2_O]^-^ at m/z 173.0099. The deprotonated molecule lost a CO_2_, forming a fragment ion [M-H−CO_2_]^-^ at m/z 147.0305. The deprotonated molecule lost H_2_O and CO_2_, forming a fragment ion [M-H−H_2_O−CO_2_]^-^ at m/z 129.0199.

The molecular formula of adenosine **(3)** is C_10_H_13_N_5_O_4_. Adenosine exhibited a protonated molecule [M+H]^+^ at m/z 268.1048 in positive ionization mode. The protonated molecule lost a C_5_H_8_O_4_, forming a fragment ion [M+H−C_5_H_8_O_4_]^+^ at m/z 136.0621.

The molecular formula of Gallic acid **(4)** is C_7_H_6_O_5_. Gallic acid exhibited a deprotonated molecule [M–H]^-^ at m/z 169.0149 in negative ionization mode. The deprotonated molecule lost a CO_2_, forming a fragment ion [M-H−CO_2_]^-^ at m/z 125.0249.

The molecular formula of phenylalanine **(5)** is C_9_H_11_NO_2_. Phenylalanine exhibited a protonated molecule [M+H]^+^ at m/z 166.0865 in positive ionization mode. The protonated molecule lost a NH_3_, forming a fragment ion [M+H−NH_3_]^+^ at m/z 149.0600. Fragment ion lost a H_2_O, forming a fragment ion [M+H−NH_3_−H_2_O]^+^ at m/z 131.1494. Fragment ion lost a HCOOH, forming a fragment ion [M+H−NH_3_−H_2_O−HCOOH]^+^ at m/z 120.0812.

The molecular formula of geniposidic acid **(6)** is C_16_H_22_O_10_. Geniposidic acid exhibited a deprotonated molecule [M–H]^-^ at m/z 373.1159 in negative ionization mode. The deprotonated molecule lost a Glc, CO_2_, H_2_O gradually, forming a fragment ion [M-H−Glc]^-^ at m/z 211.0621, [M-H−Glc−H_2_O]^-^ at m/z 193.0512, [M-H−Glc−H_2_O−CO_2_]^-^ at m/z 149.0613. The deprotonated molecule undertaking the γH reaction, forming a fragment ionat m/z 123.0456.

The molecular formula of catalpol **(7)** is C_15_H_22_O_10_. Catalpol acid exhibited a deprotonated molecule [M-H]^-^ at m/z 361.1140 in negative ionization mode. The deprotonated molecule lost a Glc, forming a fragment ion [M-H−Glc]^-^ at m/z 199.0978.

The molecular formula of chlorogenic acid **(8)** is C_16_H_18_O_9_. The protonated molecule [M+H]^+^ with a m/z of 355.0982 was detected in the positive ion mode. The deprotonated molecule [M-H]^-^ with a m/z of 353.0877 was detected in the negative ion mode. The protonated molecule lost a quinine acyl (m/z 192), forming a fragment ion [M+H−quinine acyl]^+^ at m/z 163.0391.

The molecular formula of oxidized paeoniflorin **(9)** is C_23_H_28_O_12_. Oxidized paeoniflorin exhibited a deprotonated molecule [M-H]^-^ at m/z 495.1532 in negative ionization mode. The deprotonated molecule lost a HCHO, forming a fragment ion [M-H−HCHO]^-^ at m/z 465.1406. The deprotonated molecule lost a Glc, forming a fragment ion [M-H−Glc]^-^ at m/z 333.0992.

The molecular formula of caffeic acid **(10)** is C_9_H_8_O_4_. Caffeic acid exhibited a deprotonated molecule [M-H]^-^ at m/z 179.0357 in negative ionization mode. The deprotonated molecule lost a HCOO, forming a fragment ion [M-H−HCOO]^-^ at m/z 135.0457.

The molecular formula of vanillic acid **(11)** is C_8_H_8_O_4_. Vanillic acid exhibited a deprotonated molecule [M-H]^-^ at m/z 167.0356 in negative ionization mode. The deprotonated molecule lost a HCOO, forming a fragment ion [M-H−HCOO]^-^ at m/z 123.0456.

The molecular formula of xanthotoxin **(12)** is C_12_H_8_O_4_. Xanthotoxin exhibited a protonated molecule [M+H]^+^ at m/z 217.0975 in positive ionization mode. The protonated molecule lost a NH_3_, forming a fragment ion [M+H−HCOO]^+^ at m/z 171.0919.

The molecular formula of rehmapicroside **(13)** is C_16_H_26_O_8_. Rehmapicroside exhibited a deprotonated molecule [M-H]^-^ at m/z 345.1580 in negative ionization mode. The deprotonated molecule lost a gku, forming a fragment ion [M-gku]^+^ at m/z 183.1035. The deprotonated molecule lost a C10H15O2, forming a fragment ion [M-C_10_H_15_O_2_]^+^ at m/z 179.0570.

The molecular formula of vanillin **(14)** is C_8_H_8_O_3_. Vanillin exhibited a deprotonated molecule [M-H]^-^ at m/z 151.0407 in negative ionization mode. The deprotonated molecule lost a CH_3_, forming a fragment ion [M-H−CH_3_]^-^ at m/z 136.0164.

The mass spectrometry data of the two peaks were analyzed at 8.43 min and 9.18 min. The sodium ionized molecule [M+Na]^+^ with a m/z of 503.1535 was detected in the positive ion mode. By losing the glucose and benzoic acid, forming fragment ions [M+Na−benzoic acid]^+^, [M+Na−Glucose]^+^ and [M+Na−benzoic acid−Glucose]^+^ at m/z 381.1146, 341.1006 and 219.0634. According to the polarity, it is classified as albiflorin **(15)** and paeoniflorin **(16)**.

The molecular formula of ferulic acid **(17)** is C_10_H_10_O_4_. Ferulic acid exhibited a deprotonated molecule [M-H]^-^ at m/z 193.0513, and the deprotonated molecule lost a CH3, forming a fragment ion [M-H−CH_3_]^-^ at m/z 178.0279. Moreover, the deprotonated molecule lost a HCOO, forming a fragment ion [M-H−HCOO]^-^ at m/z 149.0613. And the fragment ion [M-H−CH_3_]^-^ lost a HCOO to form the [M-H−CH_3_−HCOO]^-^ at 134.0379.

The mass spectrometry data of the two peaks were analyzed at 12.23 min and 12.94 min. The deprotonated molecule [M-H]^-^ with a m/z of 623.2012 was detectedin the negative ion mode. The deprotonated molecule lost a C_9_H_6_O_3_, forming a fragment ion [M-H−C_9_H_6_O_3_]^-^ at m/z 461.1687. According to the polarity, it is classified as verbascoside **(18)** and isoacteoside **(19)**.

The molecular formula of psoralen **(20)** is C_11_H_6_O_3_. Psoralen exhibited a protonated molecule [M+H]^+^ at m/z 187.0757 in positive ionization mode. The MS characteristics were m/z 169.0651 [M+H−H_2_O]^+^, 159.0808 [M+H−CO]^+^ and 131.0859 [M+H−2CO]^+^.

The molecular formula of dihydrosenkyunolide C **(21)** is C_12_H_16_O_2_. Dihydrosenkyunolide C exhibited a protonated molecule [M+H]^+^ at m/z 207.1021 in positive ionization mode. The protonated molecule lost a H_2_O, forming a fragment ion [M+H−H_2_O]^+^ at m/z 189.0915.

The molecular formula of senkyunolide A **(22)** is C_12_H_16_O_2_. Senkyunolide A exhibited a protonated molecule [M+H]^+^ at m/z 193.1227 in positive ionization mode at 19.62 min. The MS characteristics were m/z 175.1121 [M+H−H_2_O]^+^, 147.1171 [M+H−H_2_O−CO]^+^, 137.0600 [M+H−C_4_H_8_]^+^ and 119.0859 [M+H−C_4_H_8_−H_2_O]^+^.

**1.2 Histopathology, Masson’s Trichrome staining and immunohistochemistry**

After immobilized with 4 % PFA and embedded in paraffin, livers and intestine tissues were cut into 4.5-μm sections and stained with hematoxylin and eosin (H&E) and Masson’s Trichrome (liver only) as previously described [1]. For immunohistochemistry staining, paraffin sections were rehydrated, antigen retrieval by EDTA and blocked endogenous peroxidases with 0.3% H_2_O_2_. After blocked with BSA reagent supplemented with 10% goat serum, slides were incubated with primary antibody against ECAD (dilution, 1:200) at 4 °C overnight. After washed and incubated with goat anti-mouse/rabbit IgG HRP polymer secondary antibody (ZSGB-BIO, Beijing, China), paraffin slides were imaged by Aperio Versa (Leica, Wetzlar, Germany).

**1.3 Measurement of serum liver functional enzyme activities**

Mice were sacrificed to collect serum by centrifugation at 6000 g for 10 min. Serum levels of AST, ALT and ALP were determined using commercial colorimetric kits (Nanjing Jiancheng Bioengineering Institute, Nanjing, China) following the manufacturer's instructions of corresponding kits.

**1.4 Quantitative Real-time PCR (qPCR) analysis**

Total RNAs were isolated from mice livers and intestines by Trizol reagent and measured using NanoDrop One Microvolume UV-Vis Spectrophotometer from Thermo (Waltham, USA). cDNA was synthesized using HiScript III RT SuperMix kit according to the manufacturer’s specifications. The mRNA levels of targeted genes were quantified by qPCR as previously described [2]. List of used primers was provided in **Table S1**.

**1.5 Western blot analysis**

Liver and intestine tissues were lysed using RIPA buffer containing protease and phosphatase inhibitors. Equivalent protein was prepared and separated using 10% SDS-PAGE gels and then transferred onto the PVDF membranes. After blocked with non-fat milk, the bands were incubated with relative primary antibodies at 4 °C overnight. Finally, membranes were washed with TBST buffer, incubated with relative secondary antibodies and visualized using the ChemiDoc^TM^ Touch Imaging System (Bio-Rad, USA).

**2. Additional figure legends**


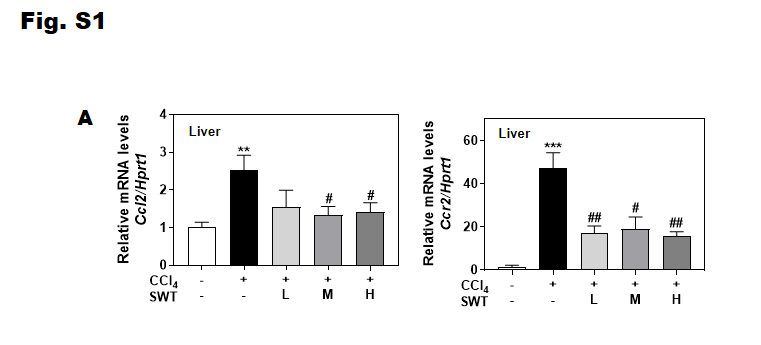


**Fig. S1 (A)** Relative mRNA levels of *Ccl2* (left panel) and *Ccr2* (right panel) in liver tissues were determined by qPCR and normalized using *Hprt1* as an internal control. Statistical significance: ***P*<0.01, ****P*<0.001, compared with control group; ^#^*P*<0.05, ^##^*P*<0.01, compared with CCl_4_ group. One-way ANOVA with Tukey’s post-hoc tests (n=8).


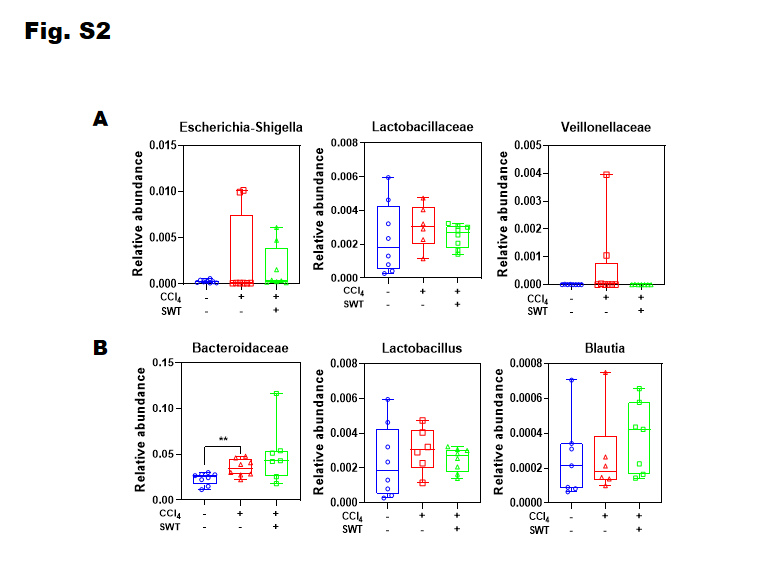


**Fig. S2 (A)** Relative abundance of *Escherichia-Shigella*, *Lactobacillaceae* and *Veillonellaceae*. **(B)** Relative abundance of *Bacteroidaceae*, *Lactobacillus* and *Blautia. **P*<0.01, compared with control group. One-way ANOVA with Tukey’s post-hoc tests (n=8).


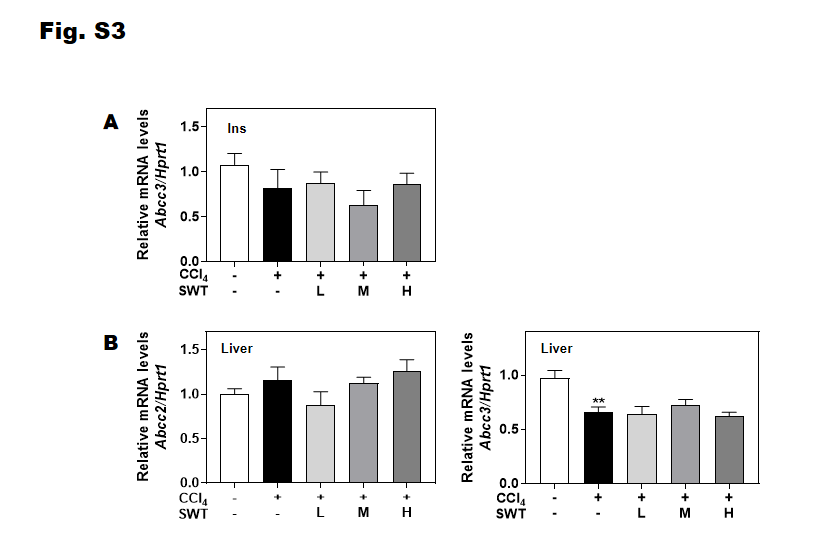


**Fig. S3** Relative mRNA level of **(A)** *Abcc3* in the intestine, **(B)** *Abcc2* and *Abcc3* in the liver were determined by qPCR and normalized using *Hprt1* as an internal control. Statistical significance: ***P*<0.01, compared with control group. One-way ANOVA with Tukey’s post-hoc tests (n=8).


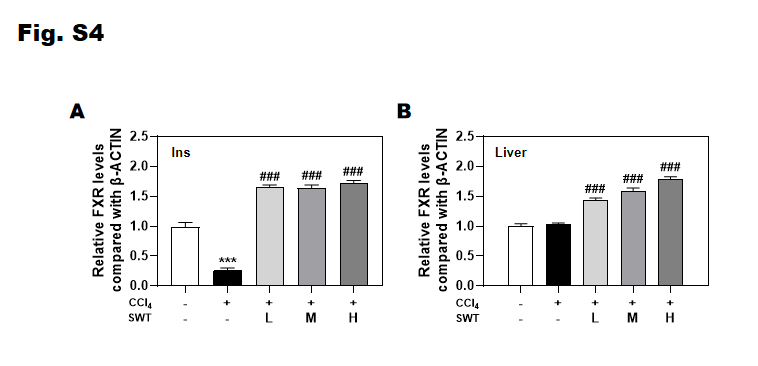


**Fig. S4** The relative density of FXR/β-ACTIN in the intestine (**A**) and liver (**B**). Statistical significance: ****P*<0.001, compared with control group; ^###^*P*<0.001, compared with CCl_4_ group. One-way ANOVA with Tukey’s post-hoc tests (n=8).

**References**

1. Li X, Liu R, Wang Y, Zhu W, Zhao D, Wang X, et al. Cholangiocyte-Derived Exosomal lncRNA H19 Promotes Macrophage Activation and Hepatic Inflammation under Cholestatic Conditions. Cells. 2020;9(1).

2. Liu R, Li X, Zhu W, Wang Y, Zhao D, Wang X, et al. Cholangiocyte-Derived Exosomal Long Noncoding RNA H19 Promotes Hepatic Stellate Cell Activation and Cholestatic Liver Fibrosis. Hepatology. 2019;70(4):1317-1335.
