# Supplementary material for: Si-Wu-Tang ameliorates fibrotic liver injury via modulating intestinal microbiota and bile acid homeostasis
Source: Chin Med. 2021 Nov 4;16:112. doi: 10.1186/s13020-021-00524-0 (PMC8570021; doi:10.1186/s13020-021-00524-0)
Supplement: Supplementary file 2 — Additional file 2: Table S1. Primer sequences used in qPCR. [file 13020_2021_524_MOESM2_ESM.docx]

| **Table S1. Primer sequences used in qPCR.** | | |
| --- | --- | --- |
| **Genes** | **Forward primer (5’-3’)** | **Reverse primer (5’-3’)** |
| *Abcb11* | TGACTACGACGTTGAGTTACAA | CTGGCAAATTTGATCATTTCGC |
| *Abcc2* | GTTTCTTCGGCATAGTTAGCAC | GGATGTAGCCACATAAAACACC |
| *Abcc3* | CCCTGCGTATGAACTTAGATC | CTGCCTCTGGCCAACACTG |
| *Acta2* | GTCATCCACAGACAGAGTAGG | CTCCCAACAGACCTGTCTATAC |
| *Ccl2* | TCCACAACCACCTCAAGCACTTC | GGCATCACAGTCCGAGTCACAC |
| *Ccr2* | GCTCATCTTTGCCATCATGATT | TCATTCCAAGAGTCTCTGTCAC |
| *Cdh1* | AGC AGT TCG TTG TCG TCA C | TCC TCG TTC TCC ACT CTC AC |
| *Col1a1* | TGAACGTGGTGTACAAGGTC | CCATCTTTACCAGGAGAACCAT |
| *Cyp7a1* | CAGAAGCATAGACCCAAGTG | GTAGCAGAAGGCATACATCC |
| *Fgfr4* | CTCGGAAAGCCCCTGGGTGA | AGCTTCATCACCTCCATCTCG |
| *Fgf15* | CTGTGTCAGATGAAGATCCACT | CAAATTTCGTTCGTTTTGGTCC |
| *Fn1* | CTATAGGATTGGAGACACGTGG | CTGAAGCACTTTGTAGAGCATG |
| *H19* | CATCCAGCCTTCTTGAAC | GGGTAGCACCATTTCTTT |
| *Hprt1* | CAGACTTTGTTGGATTTGAAA | GCTCATCTTAGGCTTTGTAT |
| *Il1b* | AATCTCACAGCAGCACATC | AGCAGGTTATCATCATCATCC |
| *Il6* | CTCCCAACAGACCTGTCTATAC | CCATTGCACAACTCTTTTCTCA |
| *Nos2* | ACTCAGCCAAGCCCTCACCTAC | TCCAATCTCTGCCTATCCGTCTCG |
| *Nr0b2* | CTCTCTTCCTGCTTGGGTTG | CAGCCAGTGAGGGTTGTG |
| *Nr1h4* | GCAACCAGTCATGTACAGATTC | TTATTGAAAATCTCCGCCGAAC |
| *Ocln* | TGCTTCATCGCTTCCTTAGTAA | GGGTTCACTCCCATTATGTACA |
| *Osta* | TCGCTTGCTCACCTCCCTACTC | ATACCCAACCTTGTCGTCTTTCCTTC |
| *Ostb* | TTCCATCCTGGTCCTGGCAGTC | GGTCTTCTGGTGTTTCTTTGTCTTGTG |
| *Slc10a1* | TCTCCCTCTCTGTGGCTGTC | TGCTGATGGTGCGTCTGC |
| *Slc10a2* | CCAATATCCTGGCCTATTGGAT | AGAGCAACCAGAGAAATACCAA |
| *Tgfb1* | GACCTCAAGAGCTCTAACATCC | GTCATCCACAGACAGAGTAGG |
| *Tnfa* | GAGAGAAAGTGAGTGCGTCCCTTG | GGCAACAGCACCGCAGTACC |
|  |  |  |
